# Prof. White

**Published:** 1851-02

**Authors:** 


*Prof. White*—Prof. W. sailed for Havre in the packet ship New York, on the 17th ult. He expects to remain abroad a year, chiefly with a view to objects connected with instruction from the Chair held by him in the University of Buffalo.

